# A report on digitised neuronal tracing method to study neurons in their entirety

**DOI:** 10.1016/j.mex.2022.101715

**Published:** 2022-04-29

**Authors:** Zeeshan Ali, G. Sivakumar, Krishnamoorthi Prabhu, Chinmay Ajit Suryavanshi, Sareesh Naduvil Narayanan

**Affiliations:** a Krupanidhi College of Physiotherapy, Affiliated to Rajiv Gandhi University of Health Sciences, Bangalore 560035, India; b Department of Physiology, Kasturba Medical College, Manipal, Manipal Academy of Higher Education, Manipal, Karnataka, 576104, India; c Department of Physiology, Ras Al Khaimah College of Medical Sciences, Ras Al Khaimah Medical & Health Sciences University, Ras Al Khaimah, PO Box. 11172, United Arab Emirates

**Keywords:** Neuron tracing, Golgi-Cox staining, Simple neurite tracer, Camera lucida tracing, Sholl analysis

## Abstract

Conventional camera lucida (CL) aided neuronal tracing technique for studying neural plasticity is a demanding procedure. Stereo Investigator-Neurolucida enabled neuronal tracing system is not accessible to all researchers. This necessitates alternate simple and less challenging digitised neuronal tracing methods. This report describes a novel digitised neuronal tracing method using widefield microscopy, and its effectiveness is compared with the traditional camera lucida aided neuronal tracing method. Golgi-Cox stained hippocampal cornu ammonis area-3 (CA3) pyramidal neuron photomicrographs were serially captured at a depth of every 2µm in the z-axis by a wide field microscope from the point of appearance to the disappearance. These images were stacked along the axis perpendicular to the image plane to reconstruct the neuron in its entirety, digitally traced and dendritic quantification was performed using open source software. The same neurons were manually traced using camera lucida, and Sholl analysis was done manually to quantify the dendritic arborisation pattern. The dendritic quantification data were not significantly different in both methods. Hence, the technology-enabled, less demanding, and equally accurate neuronal tracing can be adopted instead of manual tracing and analysis of neurons.

• A simple digitised neuronal tracing method is described.
• It is fast, rigorous, and comparable to traditional tracing techniques.
• Helps the researcher to repeatedly probe data to reduce errors.

## Graphical abstract

**Figure:**
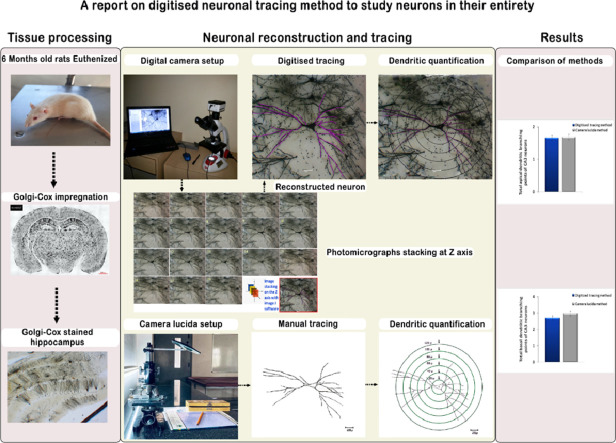

Specifications table

**Table:** 

Subject Area:	Neuroscience
More specific subject area:	*Neuron tracing, Neuron morphology, Dendritic arborisation, Neuroanatomy.*
Method name:	Semi-Automated Neuron Tracing Technique
Name and reference of original method:	*NA*
Resource availability:	Motic's digital wide-field camera microscope and freely available open source software i.e. Simple Neurite Tracer- FIJI, Image J, CorelDRAW Graphic suite (for digital Sholl's grid analysis) https://imagej.net/software/fiji/downloadshttps://www.coreldraw.com/en/?link=wm

## Background

In recent years, a lot of focus in medical research has been taking place to understand the functioning of the brain. Various methods and techniques ranging from histology to modern imaging of the brain are being used to study the central nervous system (CNS). Neuron morphology has been used as an index to assess the functioning of the brain [1]. Axonal and dendritic arbors of neurons form the backbone of the neural networks in the CNS. Reports demonstrate changes in hippocampal neuronal dendritic arborisation patterns in response to learning and experiences [2]. Dendritic morphogenesis is influenced by various factors, including several intrinsic and extrinsic mechanisms [3], [4], [5]. Neuronal morphology has also been used to study the effect of various pharmaceutical and other interventions on CNS [6]. Quantification of dendritic arborisation is a method that is frequently used to assess the extent of neuroplasticity in both experimental and interventional studies [4], [6].

Golgi-Cox staining is one of the most effective staining techniques for studying the structure of various types of neurons in the brain [7]. Santiago Ramón y Cajal pioneered the neural tracing technique using camera lucida (CL), and is extensively used in neuroscience research [8,9]. Sholl analysis is considered one of the simplest methods for quantification of dendritic arborisation patterns and has been utilised by several researchers [10,11]. However, neuronal tracing by conventional camera lucida technique is a demanding process. Another disadvantage of camera lucida tracing is that the ultimate image of the neuron obtained is a two-dimensional picture [12]. Hence the focus axis information is misplaced, and only the plane of sectioning can be seen. Moreover, errors may occur during manual neuronal tracing or during the morphometric analysis of camera lucida tracings. With the advent of technology, many computational tools have been employed to simplify and improve the pace of neuronal tracing, but, many are not free from functional limitations [13], [14], [15]. Stereo Investigator-Neurolucida enabled neuronal tracing system is the solution for this issue [16,17]. However, these types of software may not be accessible to all researchers due to multiple reasons. *1)* The cost factor; The cost of sophisticated neuronal tracing tools is exorbitant and is beyond the affordability of smaller labs*. 2)* Accessibility issues; Some highly funded labs may have such types of software (this may include public funded universities or labs) but they may not be open to all researchers from other institutions. *3)* Training; Even if there is an opportunity to use this software, a naïve researcher cannot utilize it fully as it requires extensive training and regular usage experience. This necessitates the development of simple open source automated or semiautomated digital methods for neuronal tracing and quantification of its processes. Moreover, digitally reconstructed images allow virtually unrestricted morphometric analysis. A few open source softwares like Simple Neurite Tracer, Eutectic NTS, Filament Tracer and Neuron J, were used by the researchers for neuronal tracing [18], [19]. In this report, an attempt has been made to combine wide field microscopy and open-source software to effectively and accurately trace neurons. Also, the efficacy and accuracy of the current method has been compared with that of the traditional camera lucida method.

## Materials and methods

**Animals:** Seven healthy male albino rats of Wistar strain (6 months of age), weighing 250–300 g, were obtained from Central Animal Research Facility (CARF), Manipal Academy of Higher Education, after receiving approval from the Institutional Animal Ethics Committee ***(IAEC/KMC/106/2016).*** Rats were housed in polypropylene cages measuring 41 cm × 28 cm × 14 cm. They were maintained in a 12:12 hour Light: Dark environment, in an air-conditioned room and were fed with water and standard food *ad libitum.* All animals were kept under stress-free conditions prior to euthanisation. After euthanisation, brain was carefully dissected out, stained with Golgi-Cox as described earlier [20,21]. Six neurons per rat were selected, and a total of 42 neurons were digitally reconstructed or manually traced and analysed.

### Brain sectioning and section processing

Once the stain precipitate was removed from the tissue, it was mounted onto the tissue holder of a Sledge microtome, and coronal sections of 150-µm thickness were taken from each hemisphere. These sections were transferred to slotted cassettes, which were submerged in a petri dish containing distilled water [20]. The sections were then transferred to a petri dish containing 6% sodium carbonate solution (pH 10.06) and remained to be in this solution for 20 minutes. They were dipped once in distilled water and later treated with 70% alcohol for 10 min, 90% alcohol for 15 min, and absolute alcohol for 20 min. The sections were then transferred to xylene for 20 min for clearing. All sections were mounted using DPX (distyrene, a plasticiser, and xylene), air-dried before observation [20]. Hippocampal CA3 pyramidal neurons were selected for tracing and dendritic quantification analysis [22].

### Hippocampal CA3 neuronal tracing and dendritic quantification by digitised method

**Fig. 1:**
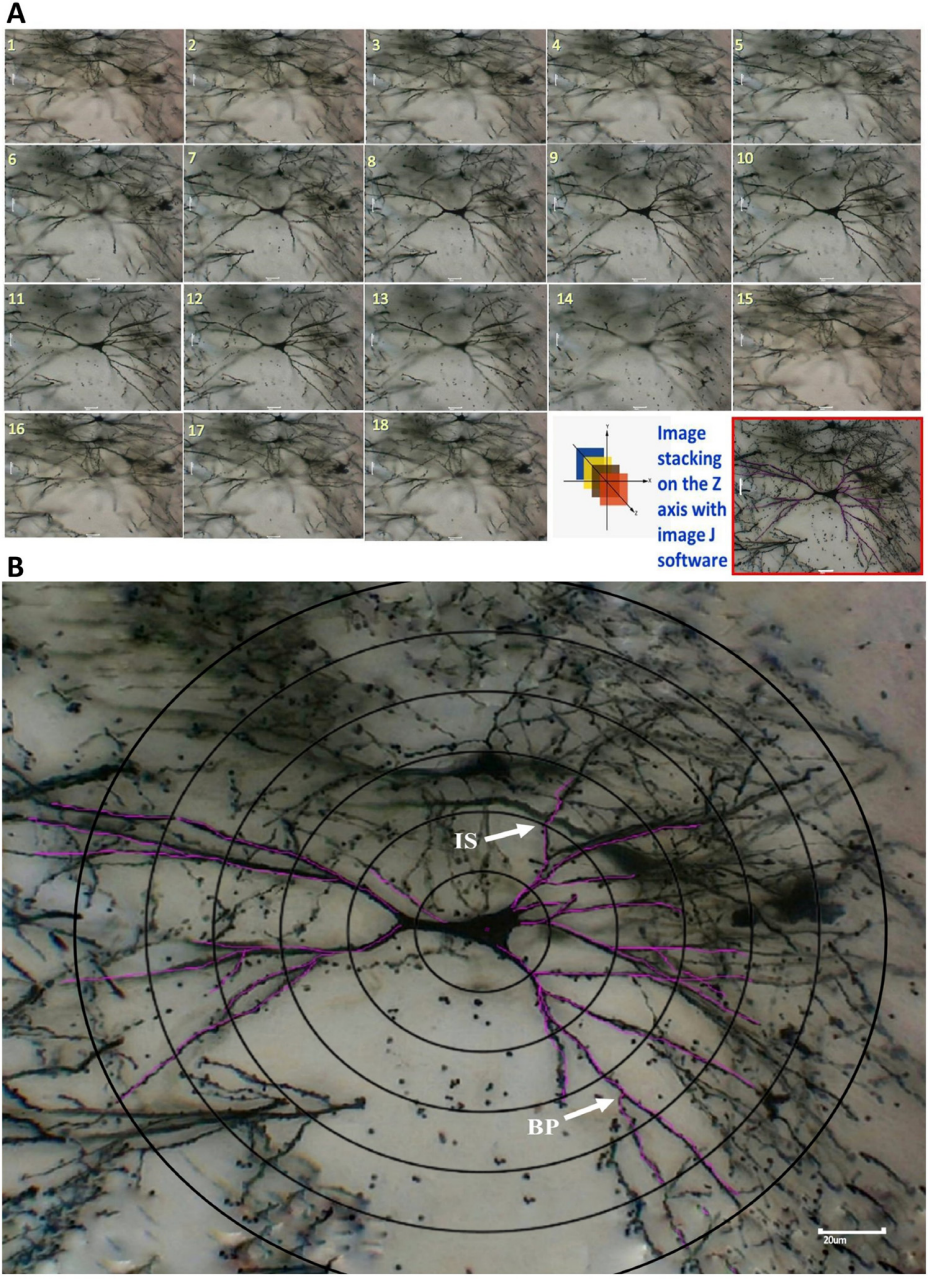
Hippocampal CA3 pyramidal neuron reconstruction (A) and computerised tracing of the same neuron (B) using open source software. (Scale: 20µm)

Photomicrographs of Golgi-Cox stained hippocampal cornu ammonis area-3 (CA3) neuron were serially captured at a depth of every ∼2µm in the z-axis by a widefield camera microscope from the point of appearance to the disappearance [23]. A widefield microscope (Motic, BA210, China), attached with a high-resolution camera (Motic, Moticam-580, China) connected to a PC installed with Image Plus 2.0 MK software, was used for image acquisition. These photomicrographs were saved in tiff format and were stacked along the axis perpendicular to the image plane to reconstruct the neuron in its entirety as a single image file (Fig. 1A and B) by FIJI software [24]. This image was used for neuronal tracing by Simple Neurite Tracer software [18]. A total of 42 neurons were traced, and their dendritic branching pattern was analysed. A single experimenter has traced all of the neurons and spent nearly 15-20 min for tracing each neuron.

### Quantification of dendritic branching points and intersections

The dendritic arborisation pattern of these neurons was quantified by performing Sholl analysis [25]. Sholl's grid was drawn using Corel Draw software [26]. A total of six concentric circles were drawn at incrementally increasing radii (20µm, 40µm, 60µm, 80µm, 100µm, and 120µm).While quantifying the dendritic branching points and intersections, this Sholl grid was superimposed on the final stacked image. The centre point of the circle was placed at the centre of the cell body as shown in Fig. 1B. Dendritic branching points (BP in Fig. 1B, a point where a dendrite diverges) and dendritic intersections (IS in Fig. 1B, where a dendrite cut through a given concentric circle) were determined in both apical and basal dendrites of CA3 neurons (Fig. 1B). Data from 6 neurons/rat was generated and the mean value for branching points (between two concentric circles) and intersections (at a given concentric circle) were calculated. From this data, the mean values of these parameters were calculated from all seven animals.

### Hippocampal CA3 neuronal tracing and dendritic quantification by camera lucida method

Initially, the simple compound microscope to which the camera lucida connected was calibrated. The measure of 1mm on the stage micrometer is equal to 100 divisions that were equal to 1000µm. The microscope was calibrated based on the spaces between the lines, such that the distance between two big lines is l00µm and that of a big and a small line is 10µm. Thus, the distance between a big and the second small line was calibrated at 20µm [27].

All 42 neurons that were traced using wide field microscopy were identified and re-traced using conventional camera lucida and quantified by Sholl analysis. The X and Y coordinates of the microscope were adjusted to identify the CA3 neurons and were traced manually by camera lucida attached to a simple compound microscope. Neurons were traced at 400× magnification with the help of a specially designed laser-tipped pencil. The fine adjustment setting in the microscope was adjusted for 0.002 mm adjustment per division. To ensure that the dendrites that have been traced from the selected neuron belong to the same target neuron, the focus is changed back and forth using the fine adjustment of the microscope at different depths. A single experimenter has traced all of the neurons and spent nearly 30 to 45 minutes to trace each CA3 neuron.

### Quantification of dendritic branching points and intersections

**Fig. 2:**
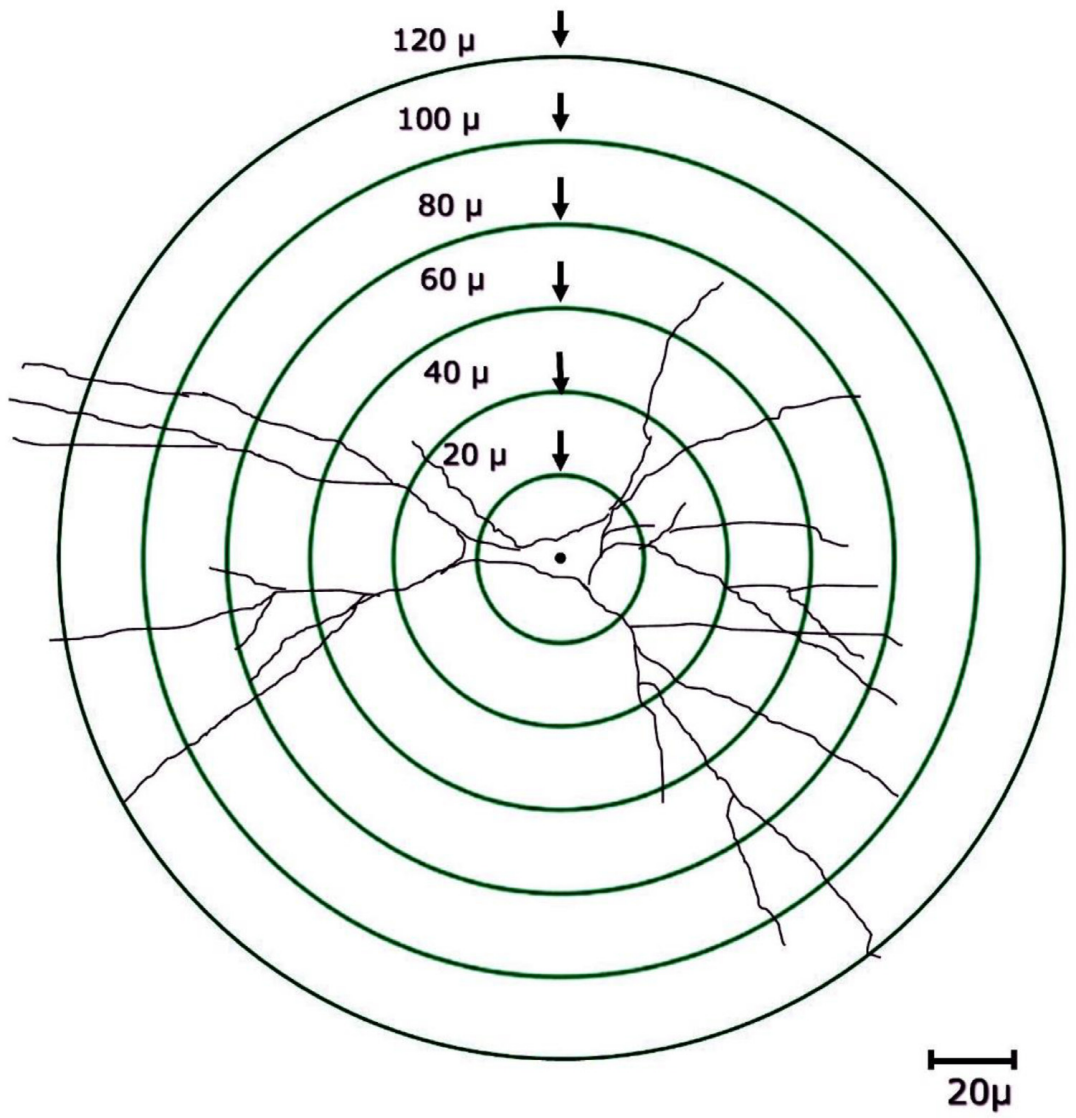
Pictorial representation of Sholl's concentric circle grids used for manual dendritic quantification of CA3 pyramidal neurons.

A stage micrometer was viewed under camera lucida at 400× magnification and the distance from the first long line to that of the second small line is marked on a paper as two dots with the help of the same laser-tipped pen. These two dots were joined to get the measurement of 20µm. Concentric circles were drawn on a transparent sheet with a radii of 20µm, 40µm, 60µm, 80µm, 100µm, and 120µm (Fig. 2) following the earlier reports [25], [28]. This sheet with the concentric circles was used for manual dendritic quantification as described by Sholl [25]. Briefly, the center point of the circle was placed at the center of the soma of a neuron as shown in Fig. 2. The branching points between two successive concentric circles (within each successive 20µm radial sphere) were manually quantified. The dendritic intersection, which is the point where the dendrite touches or intersects the given concentric circle was also manually counted [20,10]. As described earlier, data from 6 neurons/rat was generated and the mean value for branching points and intersections was calculated. From this data, the mean values of these parameters were calculated from all seven animals and this mean was compared with the data generated by digitised tracing method.

### Statistical analysis

The data are expressed as Mean±SE. The apical and basal dendritic intersections and branching points of the individual neurons for each rat were quantified, and the mean was calculated in both methods. These were compared using the independent Student's‘t’ test, and a p value ≤ 0.05 was considered significant. Statistical Package for the Social Sciences (SPSS) software was used for data analysis.

## Results

### Comparison of hippocampal CA3 apical dendritic branching points and intersections between digitised and manual tracing methods

**Fig. 3:**
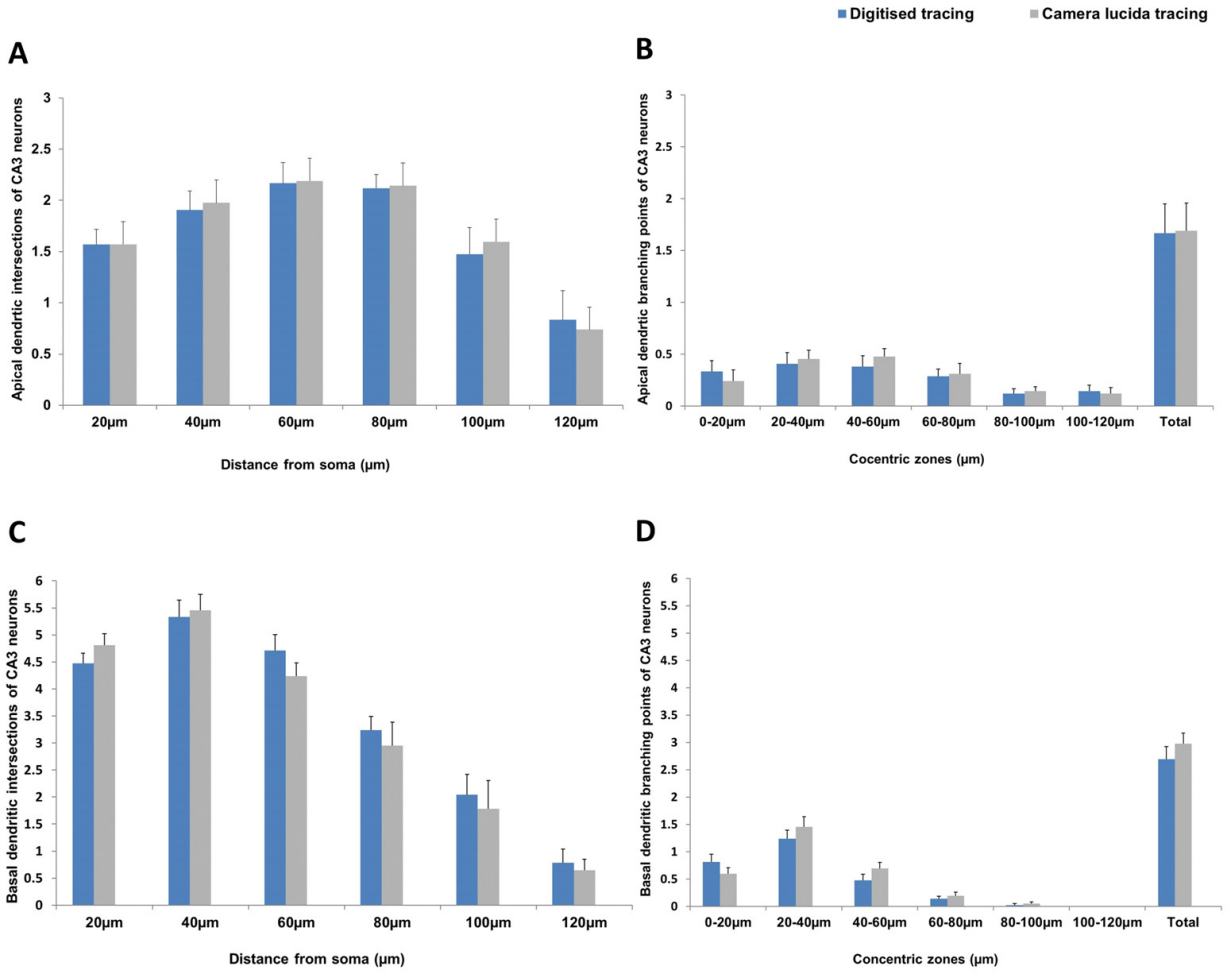
Hippocampal CA3 pyramidal neuron apical dendritic intersections at different distances from the soma (A), CA3 pyramidal neuron apical dendritic branching points at different concentric zones (B), CA3 pyramidal neuron basal dendritic intersections at different distances from the soma (C) and CA3 pyramidal neuron basal dendritic branching points at different concentric zones (D) in digital and manual tracing. [Note: Blue- Digitised tracing by open source software, Grey- Manual tracing by camera lucida.]

There was no significant difference in the apical dendritic branching points and intersections between the two methods. Although the difference was not statistically significant, the mean apical dendritic branching points of the given CA3 neuron at all levels (except at 0-20µm) was more when quantified with the traditional method compared to digitised method (Fig. 3A and B). Specifically, the CA3 apical dendritic intersections quantified with traditional method were marginally higher compared to those in the digitised method (Fig. 3A and B). Contrarily, the number of apical dendritic intersections quantified at the periphery (at 120µm) was less in traditional method compared to the same when quantified by digitised method (Fig. 3A and B). The total apical dendritic branching points of the CA3 pyramidal neurons were also found to be slightly higher when quantified using the traditional method compared to the other (Fig. 3B).

### Comparison of hippocampal CA3 basal dendritic branching points and intersections between digitised and manual tracing methods

The mean number of CA3 basal dendritic branching points and dendritic intersections quantified using digitised method were not significantly different from those that were quantified using the traditional method. However, the CA3 neuronal soma and all its proximal basal dendrites were better visualised through widefield microscopy compared to the other. Although not statistically significant, the mean number of CA3 basal dendritic branching points at all levels (except at 0-20µm) was more in camera lucida traced neurons compared to the other (Fig. 3C and D). Similarly, the CA3 basal dendritic intersections quantified using the traditional method were marginally high near soma and at early grids (20μm and 40μm) compared to those in the digitised method (Fig. 3C and D). The total basal dendritic branching points of the CA3 pyramidal neurons were also found to be slightly higher when quantified using the traditional method compared to the other (Fig. 3D).

## Discussion

Critical analysis of the results of the current study indicates that there was no significant difference between the CA3 dendritic parameters quantified using the current method (digitised method) and the traditional manual method (camera lucida method). Although not statistically significant, the number of dendritic branching points and intersections of the CA3 neurons were consistently more when quantified using the traditional method compared to the current method.

The differences in data between both methods could be due to multiple reasons. It could be due to a possible error in the dendritic tracing in case of camera lucida aided method, where the researcher was unable to differentiate the origin and continuity of a dendrite from the original parent neuron at a particular focus, which resulted in tracing of dendrites that may be originally a part of the neighbouring neurons [29]. One of the technical causes of this error could be insufficient illumination of the camera lucida microscope compared to the widefield microscope. The microscope used for digitised tracing has an inbuilt illumination adjustable neon/LED lamp source, which enables the researcher to trace the dendrites effectively compared to the one in camera lucida [30]. The other reason could be the reduced visual acuity of the researcher during dendritic tracing as a result of the serial arrangement of narrow prism with the eyepiece of camera lucida-connected microscope compared to the digitised tracing. The prolonged strain on the human eye in manual tracing could have resulted in reduced differentiation of cross-over dendrites, which were incorrectly interpreted as the branch of the same neuron and could have resulted in false high values compared to the digitised method [31]. As described earlier, in the current method, neuronal tracing and quantification were performed on high definition (HD) magnified photomicrographic images with brightly and clearly illuminated background on a laptop screen. As a result, each crossed dendrites from neighbouring neurons and the actual dendritic branching points can be clearly differentiated with their stacking order at different depths, which enables the researcher to trace the neuronal arbor easily.

Also, the real-time, prolonged, focused human observation of a neuron and its branches through narrow eyepiece-prism with a light source from underneath would possibly generate positive after images in the traditional manual tracing method [29], [32],33]. As a result, the researcher may report or obtain false tracings compared to digital photomicrography based neuronal tracing. The possibility of an error in manually quantifying dendritic arbor using transparent sheets drawn with Sholl's concentric circles cannot be ruled out in traditional methods. This can be significantly reduced in a method that uses digital platforms to quantify the dendritic arbor as described in the current method, which is one of the advantages of this method. Furthermore, the Golgi-Cox-stained slides may also have a few artefacts due to dust, mounting medium and stain residues [14], which the experimenter would wrongly consider as a part of dendritic morphology leading to false-positive results in the manual tracing method. However, these can be comfortably differentiated from dendritic arbors in high-definition photomicrographs and could be consciously ignored when viewed on a computer screen during digital tracing and quantification. Tracing techniques have evolved over the years, from pencil drawings using camera lucida to a digitising tablet that logs the tracing coordinates, followed by semiautomated methods with a computer interface and automatic algorithm-generated reconstructions that minimize manual intervention. Conventional camera lucida aided neuronal tracing technique has not changed significantly or markedly over the years. However, there are a few advancements with the light source and tracing with laser tip pencil along with a few magnification modifications [15,[33], [34].

As stated earlier, the time required for neuronal tracing and quantification can be substantially reduced by following the current method. The opportunity for re-evaluation of a manually traced neuron is extremely limited in a traditional neuronal tracing method. However, in the current method, the captured and stacked photomicrographs can be probed repeatedly to minimise errors as per the experimenter's wish and need, which further substantiates the efficacy and advantage of the current method**.**

Like any other digitised methods, there are a few limitations to the current method, which need pondering while following this method for neuronal tracing. The serial photomicrographs of neurons used to reconstruct neurons were captured at different depths (2µm apart) on the Z-axis. As they are not real-time images, during an offline dendritic quantification, the researcher is technically blind along the Z-axis between the spaces of 2µm. However, this can be effectively minimised by obtaining as many as photomicrographs on the Z-axis, which reduces the depth (possibly by less than 1µm) as per the brain area and approximate thickness of the dendrites being studied. Although the open-source software used in this study has been cited by several researchers [14], [35], the accuracy of this cannot be completely commented on at this stage because it has not been compared with the Neurolucida or related standard tracing platforms due to technical difficulties in the current study and this may be a lead for future research.

## Conclusion

As evident from the results, the dendritic quantification data were not significantly different in both methods. Hence, the technology enabled, less demanding, and equally accurate neuronal tracing method can be adopted instead of manual tracing and analysis of neurons. This significantly reduces experimenter bias and saves time for the researchers. The opportunity for re-evaluation of captured and stacked photomicrographs as per the experimenter's wish and need further substantiate the efficacy and advantage of the current digitised neuronal tracing method. A future study that compares the current digitised method with that of the newest digitised neuronal tracing tools or methods would help to reaffirm the validity of the current method further.

## Declaration of Competing Interest

The authors declare that they have no known competing financial interests or personal relationships that could have appeared to influence the work reported in this paper.
